# Transcriptome and histological analyses on the uterus of freckle egg laying hens

**DOI:** 10.1186/s12864-023-09828-x

**Published:** 2023-12-05

**Authors:** Guochao Duan, Wei Liu, Haixia Han, Dapeng Li, Qiuxia Lei, Yan Zhou, Jie Liu, Jie Wang, Yuanjun Du, Dingguo Cao, Fu Chen, Fuwei Li

**Affiliations:** 1 Poultry Institute, Shandong Academy of Agricultural Sciences, Jinan, 250100 China; 2 College of Veterinary Medicine, Qingdao Agricultural University, Qingdao, 266109 China; 3 Poultry Breeding Engineering Technology Center of Shandong Province, Jinan, 250100 China

**Keywords:** Freckle eggs, Uterus, Immunoreaction, Transcriptome, Stress response

## Abstract

**Background:**

In this study, we explored the characteristics and causes of freckle formation. We collected 15 normal and freckled eggs each for eggshell index testing and hypothesized that the structure and function of the uterus would have a direct effect on freckled egg production given that eggshells are formed in the uterus. To test this hypothesis, we collected uterine tissue from laying hens (418 days of age) that laid normal (Group C, *n* = 13) and freckled (Group T, *n* = 16) eggs for 7 consecutive days.

**Results:**

When we examined the eggshell quality, we found that the L value was significantly lower (*P* < 0.05) in the freckled site group of freckled eggs compared to the normal egg group during the detection of blunt pole, equator, and sharp pole of the eggshell color. The a-values of the three positions were significantly higher (*P* < 0.05) in the freckled site group of freckled eggs, and the a-values of the blunt pole were significantly lower (*P* < 0.05) in the background site group of freckled eggs, compared to the normal egg group. The b-values were significantly higher (*P* < 0.05) at three locations in the freckled site group of freckled eggs compared to the normal egg group. During the detection of eggshell thickness, the blunt pole was significantly higher (*P* < 0.05) in the freckled egg site group of freckled eggs compared to the normal egg group, and there was no significant difference between the other groups (*P* > 0.05). There was no significant difference (*P* > 0.05) between the transverse and longitudinal diameters of the eggs in each group.We then performed histopathology and transcriptome analyses on the collected tissue. When compared with group C, uterine junctional epithelial cells in group T showed significant defects and cilia loss, and epithelial tissue was poorly intact. From transcriptomics, genes that met (|log2FC|) ≥ 1 and *P* < 0.05 criteria were screened as differentially expressed genes (DEGs). We identified a total of 136 DEGs, with 101 up- and 35 down-regulated genes from our RNA-seq data. DEGs identified by enrichment analyses, which were potentially associated with freckled egg production were: *IFI6*, *CCL19*, *AvBD10*, *AvBD11*, *S100A12*, *POMC*, and *UCN3*. Gene ontology (GO) and Kyoto Encyclopedia of Genes and Genomes (KEGG) pathway enrichment analyses showed that pathways were associated with immunoreaction and stress stimulation, e.g., complement activation, interleukin-1 cell reactions, viral responses, cell reactions stimulated by corticotropin releasing hormone, steroid hormone mediated signaling pathways, staphylococcal infections, B cell receptor signaling pathways, and natural killer cell mediated cytotoxicity.

**Conclusions:**

From these data, freckled areas deepen freckled eggshell color, but background areas are not affected. At the same time,we reasoned that freckle eggs may result from abnormal immune responses and impaired uterine functions induced by stress. Therefore, the uterus of laying hens in a state of stress and abnormal immune function can cause the appearance of freckled eggs.

**Supplementary Information:**

The online version contains supplementary material available at 10.1186/s12864-023-09828-x.

## Introduction

For centuries, eggs have been an important staple of protein intake, thus egg quality is important for modern diets. The brown color of the eggshell is a criterion for evaluating the quality of eggs. Eggs with freckles can lower this criterion, leading consumers to doubt the quality of eggs and ultimately leading to a drop in sales. Therefore, from an economic point of view, it is important to ensure the quality of the eggshell.

On eggshells, freckles are an uneven pigmentation. Pigmentation uniformity on eggs reflects eggshell quality, which is important to many consumers [1]. In recent years, it was shown that eggshell color not only affects consumer sensory experiences, but is also relates to egg resistance to microbial invasion. For brown eggs, the degree of pigmentation affects the concentration of antifungal proteins and lysozyme [2]. Antioxidant and UV radiation resistance of eggshell pigments may also affect chicken embryo development [3, 4]. These observations suggest that eggshell pigmentation affects yield and performance in productive brown-laying hens, and that the color quality of freckled eggshells may have a profound effect on the value of the egg.

Some egg products, such as quail eggs, have a laying time similar to that of an egg [5]. Quail eggs are usually covered with large spots, which are normal compared to freckled eggs. The spots on quail eggs have been analyzed experimentally and found to have the same pigmentation as the brown eggshell. The main pigment is protoporphyrin IX [6]. For the same egg, but different eggshell color of white shell eggs and brown shell eggs compared to the main color differences are also different pigment substances [7], not only that, the quality of the egg is reflected through the eggshell color, and the eggshell color is affected by a variety of factors, such as genetics, nutrition, disease, drugs and stress [8]. These factors directly affect the physiological state of laying hens and eggshell pigmentation. Freckles are more likely to appear on brown-shelled eggs than on eggshells of other colors. Brown eggs are mainly composed of pigments such as protoporphyrin IX [9]. Currently, there are two prevailing views on the manner in which the site of pigment synthesis occurs in brown eggs: one view is that the pigment is synthesized by red blood cells in the circulatory system [10]; the other view is that the pigment is synthesized by epithelial cells in the uterus [11, 12]. Although the site of pigment synthesis is controversial, studies have shown that brown eggshell pigmentation occurs in the uterus, and the same quail uterine section was found to deposit a large number of pigment particles in the uterus during the laying period [13], so that maintaining healthy physiology of the uterine sector promotes a more homogeneous pigmentation. Therefore, we hypothesized that the physiological state of the uterus may directly influence the formation of freckled eggs.

RNA sequencing (RNA-Seq) is a useful tool for analyzing total gene expression changes between individuals with different phenotypes [14]. For poultry, many RNA-Seq havs been conducted at different physiological developmental stages and tissue sites to identify genes associated with different phenotypes [15]. However, transcriptome-related studies on eggs with freckles are very scarce.

Freckles reduce the quality of eggshell color, and we tried to find out why freckled eggs form.We sought to characterize the causes of freckled egg formation. The main difference between freckle and normal eggs lies in pigmentation uniformity. As the uterus is the main location where pigmentation primarily occurs, we hypothesized that changes in uterus structure and function generate freckle eggs. To verify this, we conducted histopathology and transcriptome analyses on the uterine tissue of laying hens thatproduced freckle and normal eggs. Although there are fewer studies on freckled eggs, previous studies have found that the pigmentation in the shells of brown eggs can affect consumers' evaluation of the quality of the eggs, and has an effect on the antimicrobial properties of the eggs. Therefore, we hope to explain the basic mechanism of freckle production and provide a theoretical basis for future research on freckled eggs and the reduction of freckled egg production.

## Material and methods

### Animal and sample collection

**Fig. 1:**
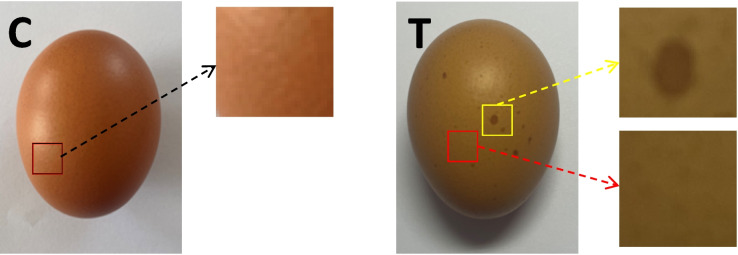
Representative images of a normal and freckle egg. C is uniform in color while the normal egg T has dark brown spots on its eggshell—freckled egg. Note: Black box: normal eggshell area. Yellow box: freckled area of freckled eggshell. Red box: freckled eggshell background area

All study animal procedures were reviewed, approved, and supervised by the Animal Ethics committee of Shandong Academy of Agricultural Science (Permit No: 2018412). Experimental chickens were collected from Juxian New Era Breeding Chicken Farm in Shangdong province. The study used 8000 Hy-line browns aged 418 days who were raised following feeding guidelines for this breed. After evaluating egg quality for 2 weeks, 16 chickens who laid freckle eggs for 7 consecutive days (T) and 13 chickens who laid normal eggs (C) were selected and grouped. Freckled eggs are shown in Fig. 1. Fifteen normal and freckled eggs each were collected for testing of relevant egg items. Experimental birds were euthanized at 7–8 h after oviposition. Euthanasia was performed by cervical dislocation, with all efforts made to minimize suffering. A 1.5 cm length of uterine tissue in the middle of the uterus was removed for histological observations and transcriptome analyses. Uterine tissue for histopathology was rinsed in normal saline and fixed in 10% formaldehyde. Tissue for transcriptome analysis was rapidly frozen in liquid nitrogen and stored at-80 °C.

### Eggshell quality test

Fifteen normal and freckled eggs each were divided into three groups according to the experimental site: normal eggs, freckled eggs with freckled areas, and freckled eggs with background areas. For the eggshell color measurement of each group, the blunt pole, the equator and the sharp pole of the three groups were examined using a colorimeter CM-700D (Konica Minolta CoTokyo, Japan), and the data were counted using Lab color evaluation. For the test of eggshell thickness in each group, the blunt pole, equator and sharp pole of the eggshells of the three groups were selected, the internal egg membrane was removed, and the test was performed using the Egg Shell Thickness Measuring Ruler DPMA3 (YaoDPeng Technology Co, Beijing, China). When testing the longitudinal and transverse diameters of the eggs, the eggs were divided into normal and freckled groups, and the maximum longitudinal and transverse diameters of the eggs were tested by using electronic vernier calipers IP54 (YAODPENG Technology Co, Beijing, China).

### Histopathology on uterine tissue

Uterine tissues from laying hens of normal (C, *n* = 13) and freckled (T, *n* = 16) egg groups were fixed in formalin fixative for 48 h, rinsed with saline and trimmed.. The tissue was dehydrated in ethanol and embedded in paraffin. Paraffin blocks were sectioned into 5 μm thick slices and stained using a hematoxylin and eosin (HE) staining kit (Servicebio Technology Co. Ltd., Wuhan, China). Other slices were twice immersed in xylene for 20 min, twice in ethanol for 5 min, in 75% ethanol for 5 min, and rinsed at least three times in distilled water. Each slice was stained in hematoxylin for 5 min, and sequentially immersed in 1% hydrochloric acid and 0.8% ammonia solution. After this, slices were rinsed in distilled water for 5 min, and sequentially dehydrated in 85% and 95% ethanol. After staining in eosin for 10 min, slices were rinsed in distilled water for 5 min. Then, slices were washed three times in ethanol for 5 min each time, and twice in xylene for 5 min each time. Slices were sealed with neutral colloid and preserved for histomorphological observations. Finally, HE tissue observations were conducted using a Nikon YS 100 microscope (Nikon Corporation, Tokyo, Japan) equipped with digital camera at 40 × magnification. We assessed uterine epithelial tissue integrity and villi attachment.

### Total RNA isolation and cDNA library construction and sequencing

RNA was extracted and purified from the uterine parts of chickens in the normal (C, *n* = 13) and freckled (T, *n* = 16) egg groups using TRizol (Invitrogen, California, USA) according to the manufacturer's protocol. Quality control of RNA quality and purity was performed using a NanoDrop ND-1000 (ThermoFisher, MA, USA). RNA integrity was also assessed using the Bioanalyzer 2100 (Agilent, CA, USA). Next, using two purification rounds, specific mRNA was captured by PolyA using oligo(dT) (Invitrogen, CA, USA). Captured polyA mRNA was fragmented at high temperature using the NEBNext® Magnesium RNA Fragmentation Module (NEB, MA, USA). Then, first strand cDNA was synthesized from segmented RNA using the Invitrogen SuperScript™ II Reverse Transcriptase kit (Invitrogen, CA, USA). RNA was removed from the cDNA-RNA hybrid to generate double-stranded cDNA using E. coli DNA polymerase I (NEB, MA, USA) and RNase H (NEB,MA,USA). Next, dUTP solution (NEB, MA, USA) was added to generate blunt ends on double-stranded cDNA. A base was ligated to the T base adaptor at both ends of the cDNA, with fragment size screened and purified using oligo(dT). Uracil-DNA glycocasylase (NEB, MA, US) was used to digest the second-strand cDNA and a library of 300 base pair (bp) ± 50 bp fragments was formed by polymerase chain reaction (PCR) amplification. Finally, Illumina Novaseq™ 6000 (Illumina, CA, USA) was used to conduct paired-end sequencing following standard operating procedures using the PE150 sequencing mode.

### Bioinformatics analysis

Raw data were stored in fastq format. We used CUTADAPT software [16] (https://cutadapt.readthedocs.io/en/stable/, version:cutadapt-1.9) to filter out unqualified sequences to generate high quality data. We then used Hisat2 [17] (https://daehwankimlab.github.io/hisat2/, version: hisat2-2.0.4) to compare clean reads with the chicken genome reference (ftp://ftp.ensembl.org/pub/release-101/fastq/gallus_gallus/dna/, version; Ensembl v101 version) and perform statistical analyses. Based on comparative results from Hisat2, Stringtie software [18, 19] (http://ccb.jhu.edu/software/stringtie/, version: stringtie-1.3.4d) was used to reconstruct transcripts and calculate gene expression levels in samples. To quantitate gene expression, Fragments Per Kilobase of transcript sequence per Millions base pairs sequenced (FPKM = [total_exon_fragments/mapped_reads (millions) × exon_length (kb)]) [20] was used as a measurable indicator in different samples. DESeq2 [21, 22] was used for differential expression analysis. Genes that met FC >  = 2 or FC <  = 0.5 ((|log2FC|) ≥ 1) and *P* < 0.05 (|log2fc|> = 1 & *P* < 0.05) criteria were screened as differentially expressed genes (DEGs) [23, 24] and Kyoto Encyclopedia of Genes and Genomes (KEGG) enrichment analyses [25] were performed on DEGs, with statistical significance set at *P* < 0.05.

### Quantitative RT-PCR analysis

High-throughput RNA-Seq DEG results were analyzed by qRT-PCR. Primer sequences were searched for in GenBank at the National Center for Biotechnology Information, and used to design and synthesize primers (Sangon Biotech, Shanghai, China). Primer sequences are shown (Additional file Table S1). Following reverse transcription, SYBR Green was used to perform qRT-PCR. For this, 2 µl of 1:10 diluted cDNA, 2 × SYBR Green Pro Tap HS Premix × 10 (Accurate Biotechnology, Hunan, China), and 0.4 μL primers were made up to 20 μL in water. Reaction conditions for quantitative real-time PCR (qRT-PCR) (Roche, Basel, Switzerland) were: 10 s at 95°C for 1 cycle, then, 5 s at 95℃ and 40 s at 60℃ for 40 cycles. Each sample was measured in triplicate and the average cycle threshold value was used for comparative analysis. The 2^−△△^Ct method was used to calculate relative gene expression levels which were normalized to β-actin.

### Statistical analysis

All analysis was performed with SPSS 24.0 software. Differ-ences between treatments were detected by Duncan’smultiple-range test and significant differences were con-sidered at *P* < 0.05. The results were expressed as mean ± SE.

## Results

### Eggshell quality test results

As shown in Table 1, during the detection of blunt pole, equator, and sharp pole of the eggshell color, the L-value of the freckled site group of the freckled eggs was significantly lower (*P* < 0.05), the L-value of the equator of the background site group was significantly lower (*P* < 0.05), and there was no significant difference in the L-value between the blunt pole and the sharp pole of the freckled egg (*P* > 0.05) compared with the normal egg group. Compared to the normal egg group, freckled eggs had significantly higher (*P* < 0.05) a-values at the three locations in the freckled site group, significantly lower (*P* < 0.05) a-values at the blunt pole background site, and no significant difference (*P* > 0.05) between a-values of the background of the equator and sharp pole sites. Compared with the normal egg group, the freckled eggs had significantly lower (*P* < 0.05) b-values at the three locations in the freckled site group, and there was no significant difference (*P* > 0.05) in the background site group. During the detection of eggshell thickness, the blunt pole of the freckled eggs was significantly higher in the freckled egg site group compared to the normal egg group (*P* < 0.05), there was no significant difference in the background site group (*P* > 0.05), and there was no significant difference in the middle and sharp pole of the eggshell thickness groups (*P* > 0.05). During the detection of transverse and longitudinal diameters of the eggs, there was no significant difference (*P* > 0.05) compared to the normal egg group.

**Table 1:** Eggshell quality test results

**Items**			**Normal egg group (*n* = 15)**	**Freckled areas in the freckled egg group (*n* = 15)**	**Background parts of the freckled egg group (*n* = 15)**
Eggshell color	blunt pole	L-value	58.72 ± 0.64^a^	51.04 ± 0.52^b^	58.19 ± 0.73^a^
		a-value	18.59 ± 0.17^b^	20.14 ± 0.26^a^	17.95 ± 0.16^c^
		b-value	28.57 ± 0.22^a^	27.25 ± 0.33^b^	28.67 ± 0.33^a^
	equator	L-value	59.95 ± 0.89^a^	52.35 ± 0.67^c^	58.77 ± 0.76^b^
		a-value	18.44 ± 0.17^b^	20.02 ± 0.21^a^	18.50 ± 0.13^b^
		b-value	28.48 ± 0.20^a^	26.94 ± 0.28^b^	28.78 ± 0.21^a^
	sharp pole	L-value	60.13 ± 0.84^a^	52.11 ± 0.52^b^	58.67 ± 0.76^a^
		a-value	18.58 ± 0.16^b^	19.68 ± 0.20^a^	18.53 ± 0.15^b^
		b-value	28.61 ± 0.22^a^	26.90 ± 0.31^b^	28.9 ± 0.21^a^
Eggshell thickness	blunt pole		0.41 ± 0.00^b^	0.44 ± 0.01^a^	0.42 ± 0.01^b^
	equator		0.43 ± 0.00	0.44 ± 0.01	0.43 ± 0.01
	sharp pole		0.44 ± 0.00	0.45 ± 0.00	0.43 ± 0.01
Transverse diameter of an egg			44.59 ± 0.23	44.58 ± 0.33	
Longitudinal diameter of an egg			58.16 ± 0.51	57.78 ± 0.94	

Means with different superscripts within the same row are significantly different (*P* < 0.05)

### Histology of uterine tissue

**Fig. 2:**
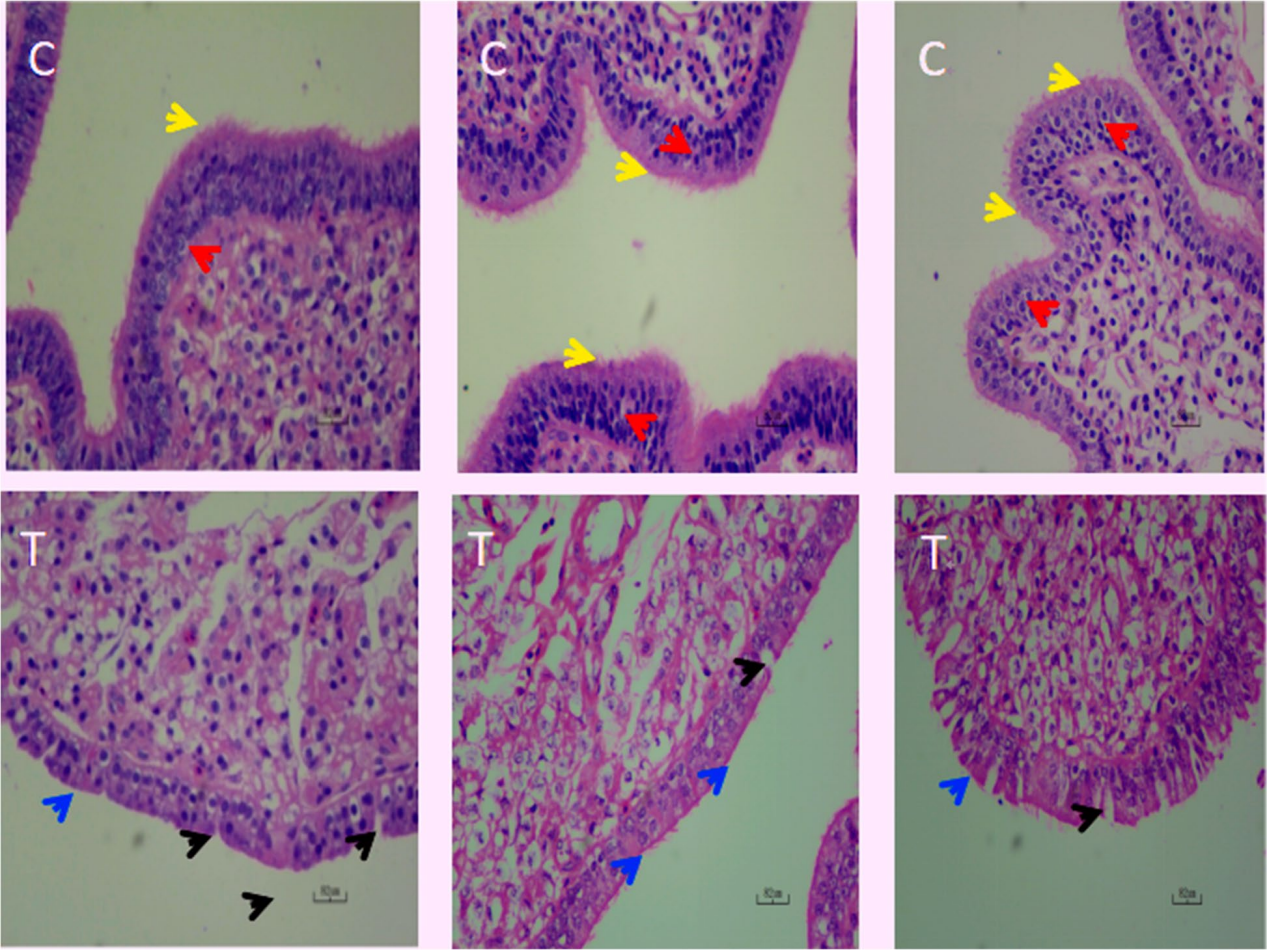
Hematoxylin and eosin staining of normal egg (group C) and freckle egg (group T) uterine tissue. (C) A representative uterine tissue sample from group C (normal eggs); (T) A representative uterine tissue sample from group T (freckle egg); red arrow—epithelial cell; yellow arrow—epithelial cell cilia; blue arrow—cilia attached to epithelial tissue has disappeared. Black arrow—defect in epithelial tissue. Images were generated under 40 × magnification

HE staining was used to analyze uterus tissue from T and C groups (Fig. 2). We observed significant differences in uterine tissue structures between groups; when compared with group C (normal eggs), group T (freckle eggs) had lower cilia density attached to epithelial tissue from the uterine part, misaligned epithelial cells, defective junctions connecting cells, and poor integrity and evenness in the uterine part.

### Quality control of transcriptome data

To identify molecular mechanisms associated with freckle egg production, RNA-Seq was performed to analyze the uterine tissue in two groups of chickens. The clean data range was between 6.47 GB and 7.42 GB and accounted for 95.01%–96.15% of raw data, respectively. Q20 values were > 99%, Q30 values were > 95%, and the GC content was 49.50%. These parameters indicated the reliability of our RNA sequences. Transcriptome data quality was shown to be reliable (Additional file Table S2), allowing for subsequent analyses.

### DEGs in the uterine tissue of chickens producing freckle eggs

**Fig. 3:**
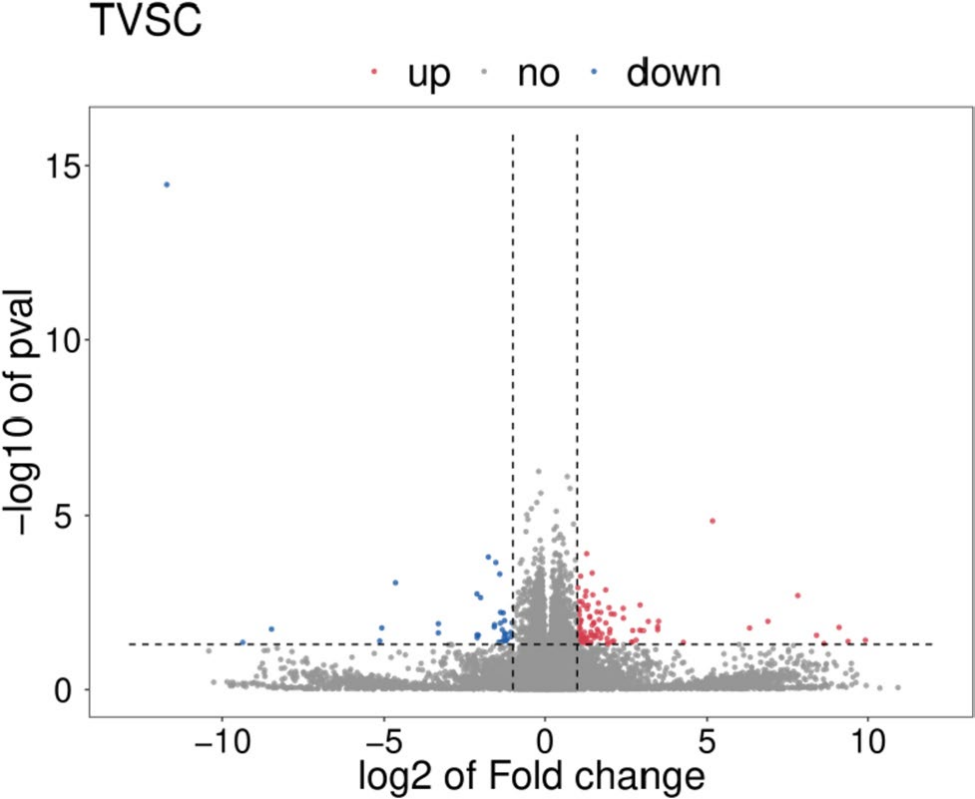
Volcano plot based on all genes in differential expression analysis. X-axis: log2(fc); Y-axis: -log10 (*P*-value); Red: up-regulated differentially expressed genes (DEGs), Blue: down-regulated DEGs, Grey spots; non-significant DEGs

**Fig. 4:**
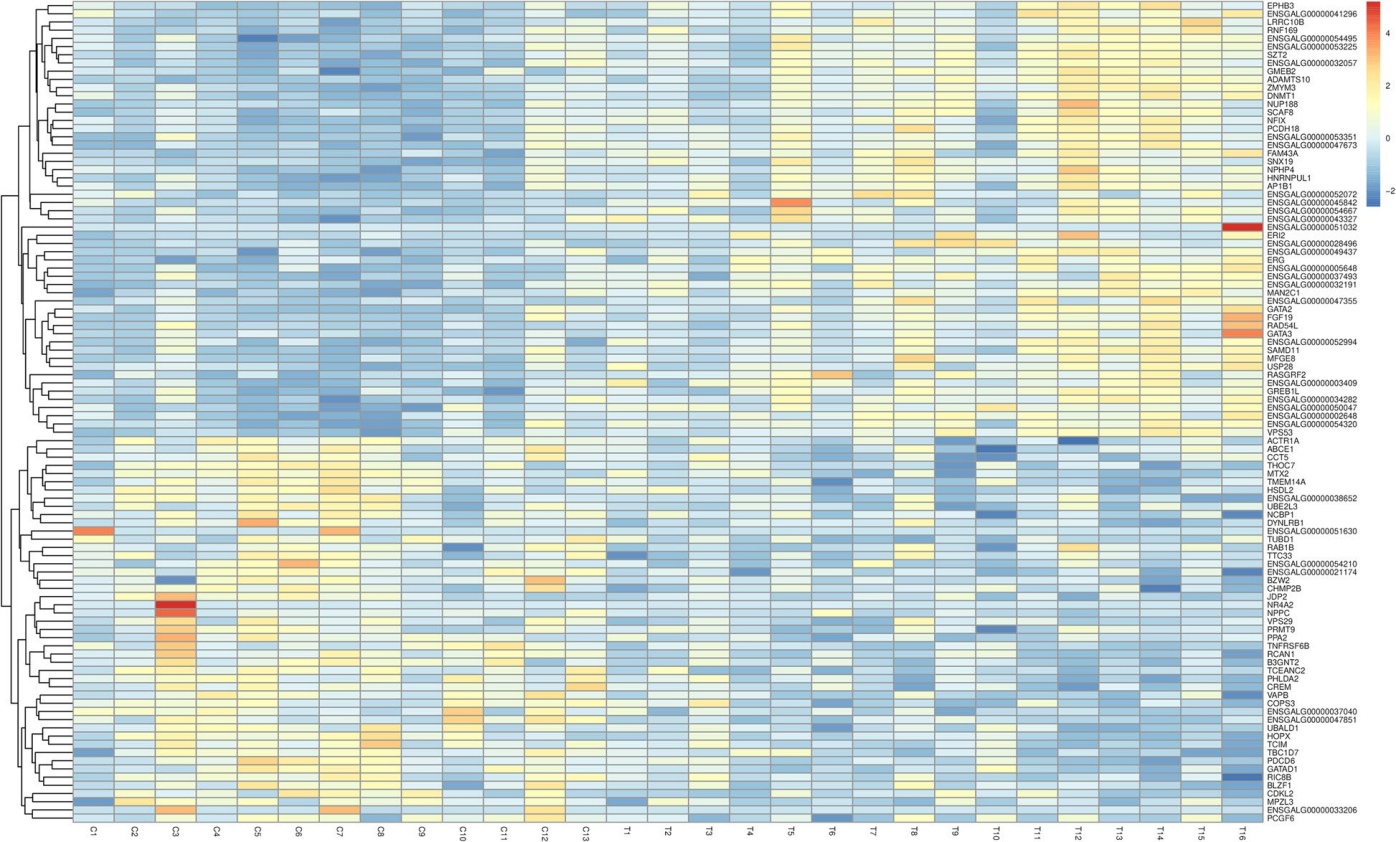
Heatmap of the top 100 differentially genes. X-axis: sample (T—freckle egg group, C—normal egg group); Y-axis: a clustering heatmap with the top 100 DEGs with the smallest default *P*-values. Red (expression pattern): up-regulated; blue (expression pattern): down-regulated

**Fig. 5:**
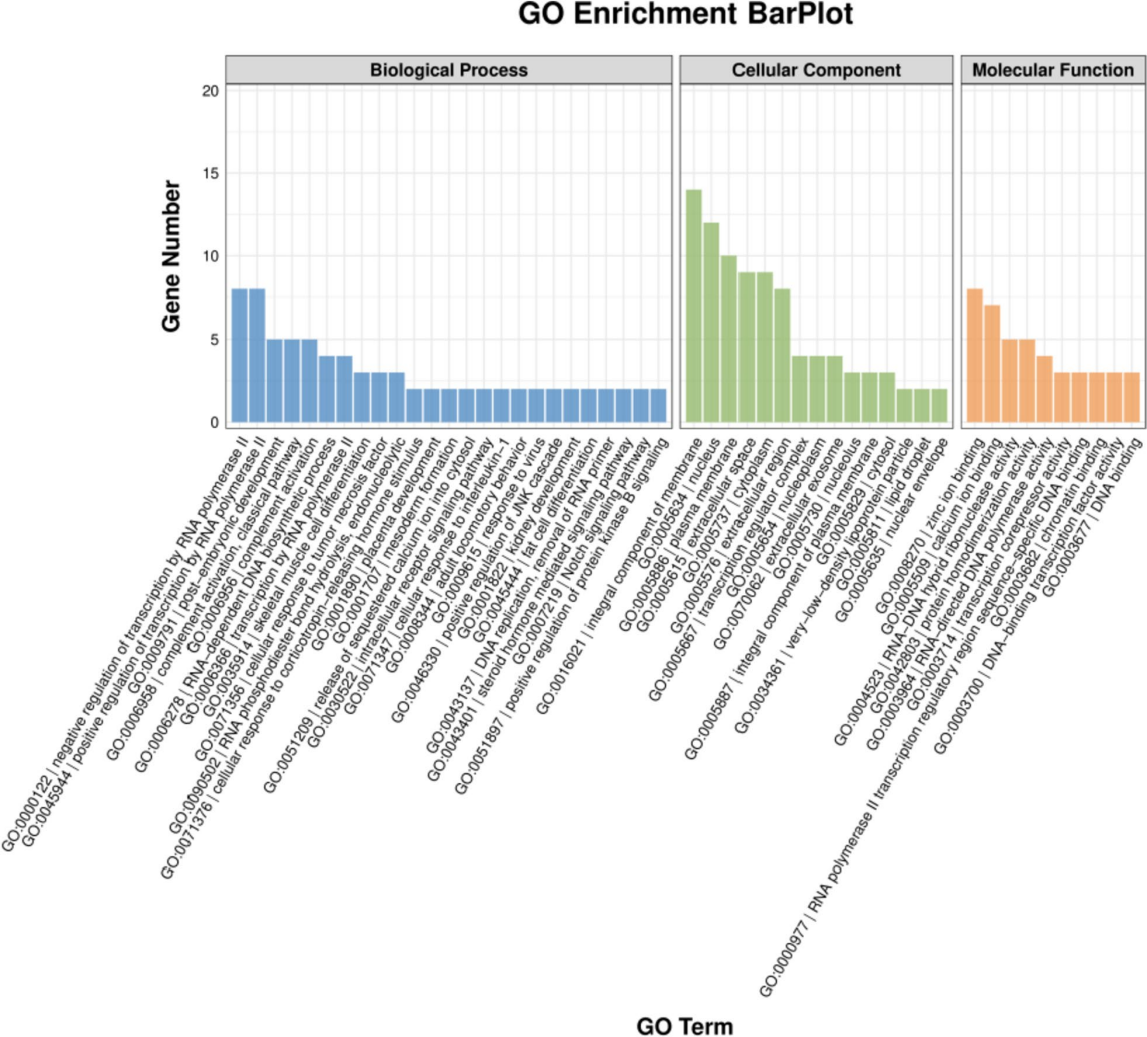
In this gene ontology (GO) enrichment analysis bar chart, differentially expressed genes (DEGs) were annotated with GO terms for biological processes, cellular components, and molecular functions. DEGs are sorted from the largest to the smallest. The top 25, 15, and 10 terms were created

In total, 21,135 genes were screened in this transcriptome analysis. When compared with the normal egg group, 136 DEGs were identified in the freckle egg group, with 101 up- and 35 down-regulated genes (Fig. 3). A heat map of the top 100 significant DEGs was created (Fig. 4). Gene expression was also quantified (Additional file Table S3). GO and KEGG pathway enrichment analyses were performed to identify potential DEG functions. In GO enrichment analysis, DEG terms were classified into three categories. The biological process category mainly contained terms associated with immunological function and embryonic growth of chickens, such as transcription regulation by RNA polymerase, DNA replication, post-blastoderm development, blastoderm development, formation of mesoderm, skeletal muscle cell differentiation, adipose cell differentiation, complement activation, cell effects of interleukin-1, viral responses, cell reactions stimulated by corticotropin releasing hormone, and steroid hormone-mediated signaling pathway. In the cellular component category, terms such as membrane component, cell nucleus, plasma membrane, and cell spaces were abundant. In the molecule function category, abundant terms included zinc binding, calcium ion binding, RNA–DNA hybrid ribonuclease activity, and DNA binding (Fig. 5 and Additional file Table S4).

**Fig. 6:**
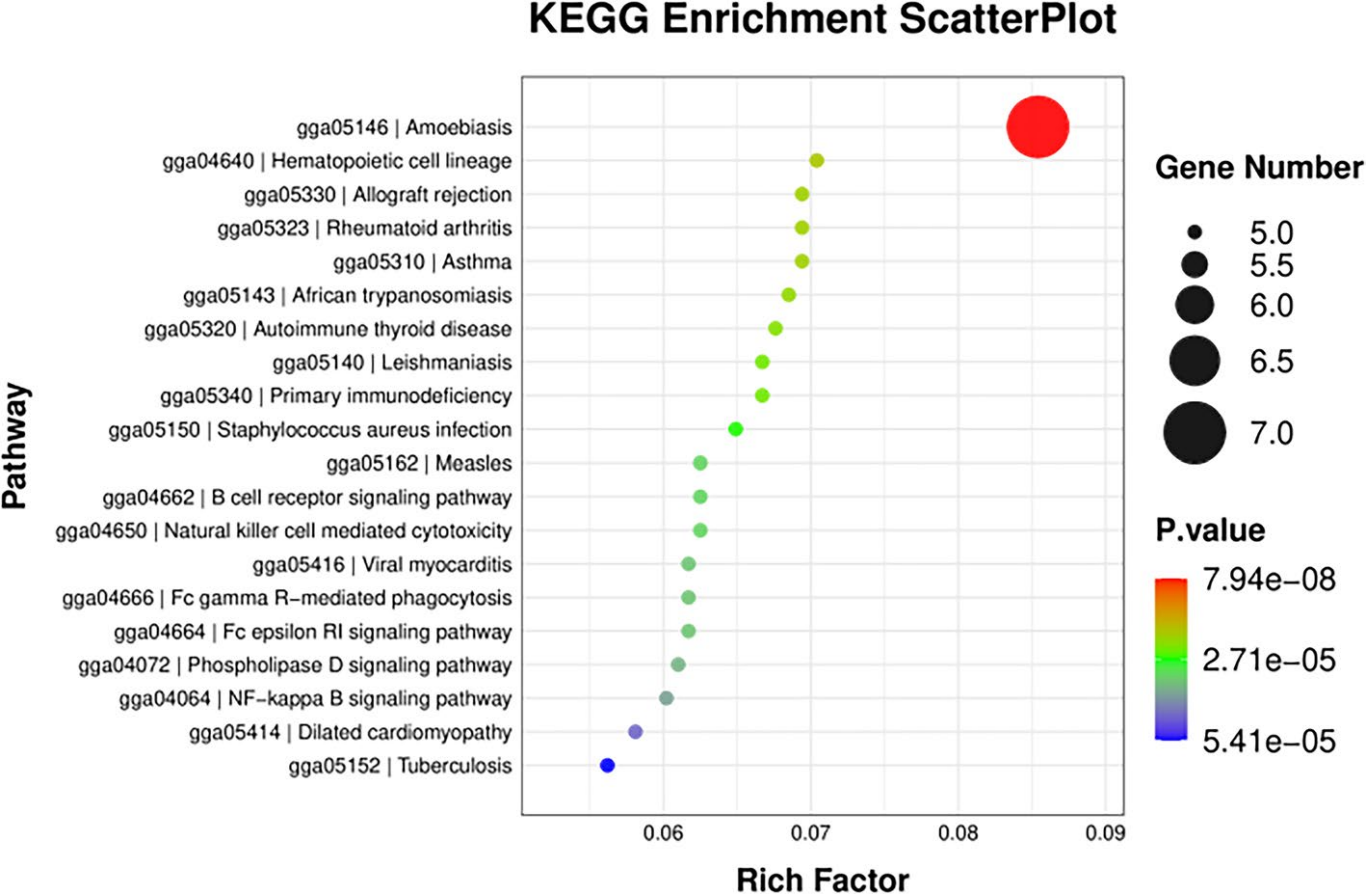
Kyoto Encyclopedia of Genes and Genomes pathway enrichment bubble chart showing the top 20 pathways with the smallest *P*-value

Several immune-related pathways were enriched in KEGG pathway analysis, including amebic protozoa, hematopoietic cell line, allotransplantation rejection, rheumatoid arthritis, asthma, African trypanosomiasis, autoimmune thyroid disease, leishmaniasis, primary immunodeficiency, staphylococcal infection, measles, B cell receptor signaling pathway, natural killer cell mediated cytotoxicity, viral myocardium, FcγR mediated phagocytosis, FcεRI signaling pathway, phospholipase D signaling pathway, and the NF-κB signaling pathway (Fig. 6, Table 2 and Additional file Table S5). Additional KEGG_Enrichment results are shown in additional table s5. From GO and KEGG results, seven candidate DEGs related to immune function and stress were screened: *IFI6, CCL19, AvBD10, AvBD11, S100A12, POMC,* and *UCN3*.

**Table 2:** Top 10 Kyoto Encyclopedia of Genes and Genomes enrichment analysis results (padj < 0.05)

Pathway_ID	Pathway	S gene number	*P*-value
gga05146	Amoebiasis	7	0.00
gga04640	Hematopoietic cell lineage	5	0.00
gga05310	Asthma	5	0.00
gga05323	Rheumatoid arthritis	5	0.00
gga05330	Allograft rejection	5	0.00
gga05143	African trypanosomiasis	5	0.00
gga05320	Autoimmune thyroid disease	5	0.00
gga05340	Primary immunodeficiency	5	0.00
gga05140	Leishmaniasis	5	0.00
gga05150	Staphylococcus aureus infection	5	0.00

### Verifying RNA-Seq accuracy

**Fig. 7:**
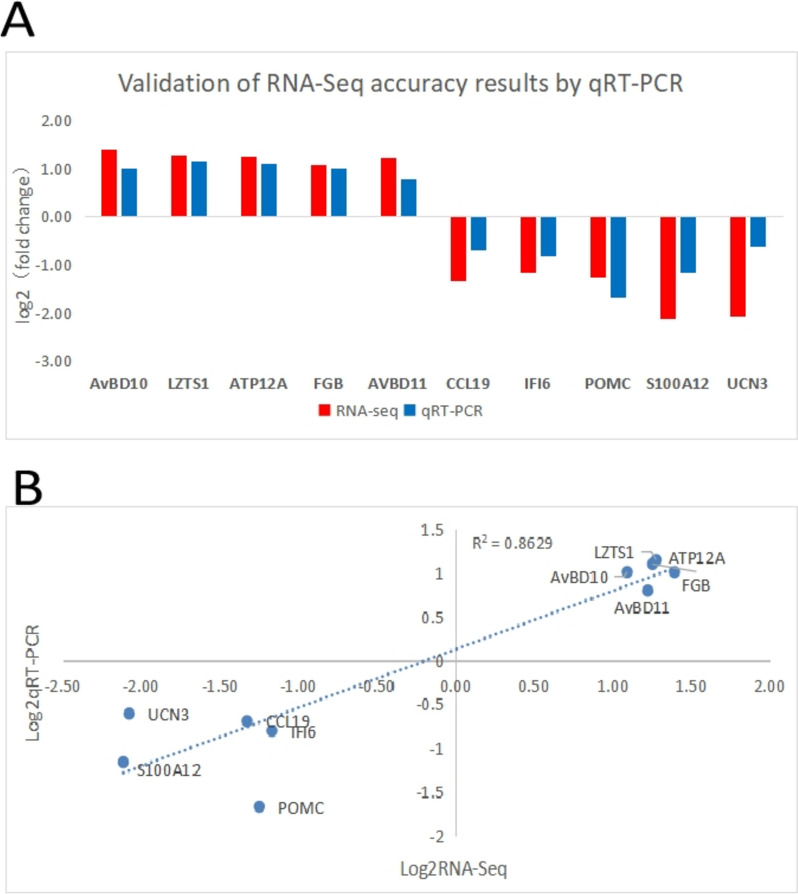
**A** Comparison of mRNA expression levels (**B**) Linear regression analysis between RNA-Seq and qRT-PCR methods

Seven DEGs potentially associated with freckled egg production were screened in RNA-Seq DEGs (*AvBD10, AvBD11, CCL19, IFI6, POMC, S100A12,* and *UCN3*), and three DEGs (*LZTS1, ATP12A,* and *FGB)* were randomly screened to validate gene expression using *β-actin* as a reference gene. All 10 genes showed consistent regulation in RNA-Seq and qRT-PCR analyses (Fig. 7A). The correlation coefficient between RNA-Seq and qRT-PCR was 0.863 (Fig. 7B), suggesting both were highly correlated and RNA-Seq was reliable for identifying DEGs.

## Discussion

Eggshell quality is associated with inheritance, nutrition, the environment, and physiological factors [26]. Freckle eggs which are eggs with dark brown spots remains one of the essential issues for quality control. The presence of freckled eggs affects the appearance of eggshells and greatly influences consumer choice. Among the effects of freckles on eggshells, the change in eggshell color is more significant compared to eggshell thickness and the longitudinal and transverse diameters of eggs. Freckles mainly reduce eggshell color quality, however, eggshell color quality is one of the most important indicators that can affect consumer choice, in order to reduce the production of freckled eggs, it is important to explore the formation mechanism of freckled eggs, however, few studies have focused on the underlining mechanisms of freckled egg formation. Because the uterus exerts a significant impact on eggshell production [8], we conducted histopathological and transcriptome analyses of the uterus in hens who produced freckled and ordinary eggs, and explored differences between groups with respect to uterus function, status, and DEGs. When compared with the normal egg group, the freckled egg group had less structural integrity of the uterus. Based on GO and KEGG pathway enrichment analyses to identify potential DEG functions, we showed that some significantly enriched GO terms were mainly related to immune function and stress responses, while from KEGG analysis, more pathways were enriched in immune function. From GO and KEGG pathway analysis, seven genes *(IFI6, CCL19, AvBD10, AvBD11, S100A12, POMC,* and *UCN3)*, possibly linked to freckle egg etiology, were screened as candidate DEGs. Importantly, although cell defects appeared in uterine tissue, no apoptosis-related DEGs were screened.

Freckles have been found to be heritable and are a moderately inherited trait [27]. In brown eggs, eggshell color is mainly regulated by pigments such as protoporphyrin IX. The deposition of eggshell pigments may have an effect on eggshell thickness, and it has been noted that freckles have an effect on the total eggshell thickness, but do not significantly enhance the effective thickness layer of their eggshells [28]. At the same time, the eggshell thickness in the freckled area of great tit eggs was rather lower than that in the background color area, which contrasts with the results of this experiment, and the effect of freckles on eggshell thickness needs to be further confirmed [29]. The higher content of protoporphyrin IX in the freckled area in the eggshell color quality caused the changes of lower L values (brightness) and b-values (blueness) and higher a-values (redness) in the freckled area [28], which caused the darker eggshell color in the freckled area.Eggshell color, which is regulated by the physiological status of chickens, may change when they are exposed to stress and pathogens [30, 31]. Eggshell color is affected by the pigmentation that occurs when the eggshell enters the uterus [32], which, as the main part of the study on eggshell color, had a direct effect on the eggshell quality, while the changes in eggshell color could reflect the health status of the uterine [33]. Newcastle disease virus (NDV) may induce uneven egg colors in chickens. In previous research, uterus histopathology, when exposed to NDV, showed atrophy, edema in epithelial cells, and also cilia fracture [34]. The uterus, if attacked by a virus, may generate fewer mitochondria in epithelial cells which could influence pigment metabolism [35]. In other research, zinc, copper, and manganese mixes supplemented to fodder appeared to enhance immune function in chickens and improve eggshell quality; histopathological observations of uterine tissue from chickens fed a zinc-copper-manganese mix improved evenness and intactness in epithelial cells, with more dense cilia [36]. These reports suggest that the physiological function and immune status of laying hens can affect uterine tissue structure and thus eggshell color quality, which is similar to the uterine tissue-induced decline in egg color quality caused by immune dysfunction in the freckled egg-producing hens in this study.

In this study, screened DEGs and pathways showed that freckle egg generation was associated with immunoreactions and stress. Related studies reported that eggshell color and quality could change if chickens were of increased age, and exposed to infectious agents and stress [37, 38]. Based on these factors and our data, we hypothesized that changes in immune functions in chickens may cause uneven eggshell color.

In a previous study, and based on brown eggshell color shades, RNA-Seq analysis was performed on liver samples and showed that DEGs were enriched in immune responses and antivirus activity [15]. These data were similar to our study which showed changes in eggshell color and immune function. Furthermore, other studies showed that anti-bacteria defensive proteins were up-regulated in response to infection and inflammation. In another RNA-Seq analysis, related genes such as AvBDs and *IFI6* displayed differential expressions, and immune molecules were differentially expressed when the immune system reacted [39].

As an important part of the immune system, defensin is a cationic antimicrobial peptide that kills bacteria and other microorganisms [40]. Previously, an RNA-Seq analysis of immune organs from waterfowl infected with NDV showed that *AVBD10* was highly positively correlated with interferon-induced *IFI6* and other inflammatory factors [41]. The expression of related immune genes in infectious bronchitis virus infected uterine epithelial cells was detected, and changes in *AvBD, CATH, IFN,* and *PGE2* expression were identified, with the defensin family showing increased significant differential expression [42]. In addition, when chickens are infected with bacteria, *AvBD10* expression increases and affects inflammatory cells, thereby providing pathogen immunity [43]. Elevated *AvBD10* and *AvBD11* mRNA differential expression levels were identified in Fayoumi chickens which were more resistant to *Eimeria coccidium* [44]. The differential expression of immune-related genes in chickens suggested the body was attacked by pathogens, tissues and organs were damaged, thus egg production was affected.

It is widely believed that eggshell color changes as chicken age. Possible causes may due to aging related immune functions deterioration in chickens, which would affect uterine pigmentation. When compared with young animals, old animals frequently experienced lower immune function in the reproductive tract and was manifested as a reduced ability of immune cells to release immune mediators [45]. The eggshell quality of old chickens decreases with increased age [46]. RNA-Seq analysis of uterine tissue from old and young chickens indicated the differential expression of a large quantity of proinflammatory genes similar to *IL-1β* [47]. Consistent with previous studies, we found that any change in immunity with age can lead to abnormal egg shell color. In our transcriptome analysis, *CCL19* and *S100A12* were important genes regulating immune responses and were significantly differentially expressed in the uterus of hens laying freckle eggs. *CCL19* and *S100A12* differential expression may indicate abnormal immune function. *CCL19* promoted chemotaxis in T cells [48]. Lipopolysaccharide is an outer cell wall component of Gram-negative bacteria and triggers immune responses [49]. When the immune system was stimulated, macrophages in chickens regulated MyD88/NF-κB signaling, causing elevated *CCL4, CCL17,* and *CCL19* expression [50]. When chickens were infected, *S100A12* was highly expressed and interacted with toll like receptor 4 to launch immune responses to remove inflammatory stimulants, thus involving systemic immune regulation [51–53]. Immune function changes in hens laying freckled eggs may cause fibrosis and atrophy of the endometrium, thus causing altered pigment metabolism, leading to abnormal eggshell pigmentation.

In our results, the stress-related differential genes *UCN3* and *POMC* were enriched in the uterine tissue of hens laying freckle eggs. Previous studies reported that stress responses increased T-NOS and MDA levels, which damaged the endometrial epithelium in laying hens and causing abnormal eggshell color. If uterine MDA levels were reduced, then oxidative stress responses would also be reduced and eggshell color quality improved [54, 55]. During the stressed state in laying hens, abnormal pigment metabolism can cause an uneven egg color [56]. When chickens were stimulated by antigens, *POMC* was down-regulated, similar to our data [57]. *UCN3* and *POMC* are regulated by the hypothalamic–pituitary–adrenal axis when chickens are subjected to certain stress stimulation, thus causing differential expression [58, 59]. Laying hens produce immune reactions under stress, metabolic disorders and redox imbalance, which affect pigmentation and produce freckled eggs.

## Conclusions

In our study of eggshell quality, freckles had a major effect on eggshell color and a non-significant effect on eggshell thickness and on the longitudinal and transverse diameters of the eggs.Our histopathology observations showed that uterine epithelial tissue integrity and villi density in the normal egg group (C) were superior to characteristics in the freckled egg group (T). From transcriptomics, 136 DEGs were enriched for immune and stress stimulation, of which seven DEGs (*AvBD10 AvBD11, CCL19, IFI6,* and *POMC, S100A12* and *UCN3)* may affect freckle egg production. In summary, we have found the cause of freckled eggs in production. When chickens are stressed and have an immune response, metabolic disturbances and redox imbalances occur, which affect pigmentation and produce freckled eggs. The next step can be used as a theoretical support point to reduce freckled eggs and improve egg shell color quality.

### Supplementary Information

**Additional file 1:** **TableS1.** Gene primer sequences were verified by QRT-PCR.

**Additional file 2:** **TableS2.** Data quality control table.

**Additional file 3:** **TableS3.** Differential Gene Expression Table.

**Additional file 4:** **TableS4.** GO pathway enrichment table.

**Additional file 5:** **Table S5.** KEGG pathway enrichment table.

## Data Availability

Raw sequence data were deposited in the Genome Sequence Archive in the BIG Data Center, Beijing Institute of Genomics, Chinese Academy of Sciences, and is publicly accessible at https://ngdc.cncb.ac.cn/gsa/s/63Psz2yy (accession no. CRA006389).
